# Genomic Scan for Selection Signature Reveals Fat Deposition in Chinese Indigenous Sheep with Extreme Tail Types

**DOI:** 10.3390/ani10050773

**Published:** 2020-04-29

**Authors:** Fuping Zhao, Tianyu Deng, Liangyu Shi, Wenwen Wang, Qin Zhang, Lixin Du, Lixian Wang

**Affiliations:** 1 Key Laborary of Animal Genetics, Breeding and Reproduction (Poultry) of Ministry of Agriculture, Institute of Animal Sciences, Chinese Academy of Agricultural Sciences, Beijing 100193, China; zhaofuping@caas.cn (F.Z.); 82101175131@caas.cn (T.D.); 82101181190@caas.cn (L.S.); lxdu@263.net (L.D.); 2 College of Animal Science and Technology, Shandong Agricultural University, Tai’an, Shangdong 271018, China; wangwenwen@sdau.edu.cn (W.W.); qzhang@sdau.edu.cn (Q.Z.)

**Keywords:** tail type, selection signature, sheep, fat deposition

## Abstract

**Simple Summary:**

According to the tail types, sheep can be briefly classified into three groups (fat-tailed, fat-rumped, and thin-tailed sheep). In this study, we used these three typical breeds from Chinese indigenous sheep breeds to perform a genome scan for selective sweeps using Ovine Infinium HD SNP BeadChip genotype data. Results showed that 25 genomic regions exhibited selection signals and harbored 73 positional candidate genes. These genes were documented not only to be associated with tail fat formation, but also be related to reproduction, body conformation, and appearance. Our findings contributed to understanding genetic basis of fat deposition in sheep tail and provide a reference for developing new sheep breeds with an ideal tail type.

**Abstract:**

It is a unique feature that fat can be deposited in sheep tails and rumps. To elucidate the genetic mechanism underlying this trait, we collected 120 individuals from three Chinese indigenous sheep breeds with extreme tail types, namely large fat-tailed sheep (*n* = 40), Altay sheep (*n* = 40), and Tibetan sheep (*n* = 40), and genotyped them using the Ovine Infinium HD SNP BeadChip. Then genomic scan for selection signatures was performed using the hapFLK. In total, we identified 25 genomic regions exhibiting evidence of having been under selection. Bioinformatic analysis of the genomic regions showed that selection signatures related to multiple candidate genes had a demonstrated role in phenotypic variation. Nine genes have documented association with sheep tail types, including *WDR92*, *TBX12*, *WARS2*, *BMP2*, *VEGFA*, *PDGFD*, *HOXA10*, *ALX4*, and *ETAA1*. Moreover, a number of genes were of particular interest, including *RXFP2* associated with the presence/absence and morphology of horns; *MITF* involved in coat color; *LIN52* and *SYNDIG1L* related to the number of teats; *MSRB3* gene associated with ear sizes; *LTBP2* considered as a positional candidate genes for number of ribs; *JAZF1* regulating lipid metabolism; *PGRMC2*, *SPAG17*, *TSHR*, *GTF2A1*, and *LARP1B* implicated with reproductive traits. Our findings provide insights into fat tail formation and a reference for carrying out molecular breeding and conservation in sheep.

## 1. Introduction

As one of the first domesticated species, sheep was probably domesticated approximately 11,000 years ago in the fertile crescent. It is documented that wild ancestors of sheep had thin tails. Then, the fat-tailed or fat-rumped sheep was bred via artificial selection [1]. Therefore, artificial and natural selection has led to an increased prevalence of fat in sheep tails across generations. Sheep can be classified into three groups according to their tail types: fat-tailed, fat-rumped, and thin-tailed [2]. Fat stored in tails or rumps acts as an energy reserve to support migration and survival during cold winters so as to adapt to hostile environments [3]. In the world, more than 25% of sheep breeds are fat-rumped or fat-tailed [4].

Domestication and artificial selection pressures have generated a series of modern sheep breeds with different phenotypic characteristics. They can adapt to a variety of environments and produce special products of meat, milk, and fine wool [2]. Selection can increase the beneficial allele frequency over time and fix it within a population, which can carve some signals in the genome. Detecting these selection signals in genomic regions is of great importance in animal genetics. These genomic regions often harbor QTLs or genes affecting economically important traits. Therefore, selection signature detection is considered as one of the strategies for identifying candidate genes. Recently, numerous studies have used genotypic data to identify selection signatures for exploring the potential genetic mechanism of phenotype polymorphisms and adaption in sheep [5,6,7,8,9].

To elucidate the genetic mechanism of sheep tail types, a handful of studies have detected selection signatures. The first genome-wide scan of selective sweeps for thin- and fat-tailed sheep was conducted by Moradi et al. [1]. They revealed QTLs on chromosomes 5, 7, and X harboring candidate genes (namely, *PPP2CA*, *SKP1*, and *TCF7*). Wei et al. [10] clustered 10 Chinese indigenous sheep breeds into two subpopulations (fat-tailed and thin-tailed sheep) and identified three strong selective genomic windows containing two functional genes (*PPP1CC* and *PDGFD*) associated with tail types. Yuan et al. [11] further employed population differentiation methods to analyze these genotypic data and found candidate genomic regions spanning 6.24 Mb with several candidate genes therein (i.e., *HOXA11*, *BMP2*, *PPP1CC*, *SP3*, *SP9*, *WDR92*, *PROKR1*, and *ETAA1*) impacting on fat tail development. Moioli et al. [12] also utilized two fat-tailed breeds vs. 13 thin-tailed breeds to perform a genome-wide scan for selection signatures and detected *BMP2* and *VRTN* genes as the most plausible genes associated with the fat-tailed phenotype in sheep. Ahbara et al. [13] divided Ethiopian indigenous sheep breeds into different groups for comparison and identified three genes (*ALX4*, *HOXB13*, *BMP4*) related to tail formation. All studies mentioned above divided many sheep breeds into two groups (fat tail and thin tail) to conduct selection signature detection. Therefore, it is hard to ignore the role of different genetic backgrounds.

**Figure 1:**
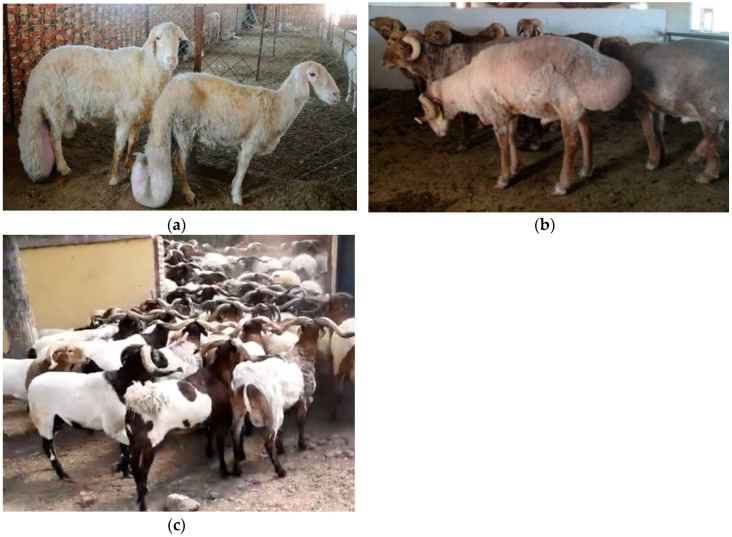
Chinese indigenous sheep breeds with three different tail types: (**a**) Large-Tailed Han sheep, (**b**) Altay sheep, and (**c**) Tibetan sheep.

In Chinese indigenous sheep breeds, three typical sheep breeds can be found. Large fat-tailed Han sheep have the largest tails (Figure 1a), Altay sheep have two large bulks of fat on the buttocks (Figure 1b), and Tibetan sheep are thin-tailed (Figure 1c). In this study, to elucidate the mechanism of tail fat deposition in sheep, we used hapFLK to detect selection signatures in these three Chinese indigenous sheep breeds with extreme tail types based on the Ovine Infinium HD SNP BeadChip (600K) genotype data. Furthermore, we annotate the genomic regions with selection signatures to explore the potential biological functions of the candidate genes under selection using bioinformatic analyses.

## 2. Materials and Methods

### 2.1. Ethnic Statement

All animal handling procedures were performed in strict accordance with the guidelines proposed by the China Animal Welfare and the Ministry of Agriculture of the People’s Republic of China. All animal experiments were approved by the Chinese Academy of Agricultural Sciences (CAAS, Permit Number: 2014-0035).

### 2.2. Genotype Data

In this study, a total of 120 individuals from three Chinese indigenous sheep breeds (40 Large-Tailed Han sheep, 40 Altay sheep, and 40 Tibetan sheep) were obtained. The information on all the individuals has been reported in our previous studies [14,15]. For the article to be self-contained, these three sheep populations are described here in detail. Large-Tailed Han sheep were collected from Liaocheng city in Shangdong Province, Altay sheep from Altay city in Xinjiang Uighur autonomous region, and Tibetan Sheep from Tianzhu county in Gansu Province. Their phenotypic characteristics and environmental variables are summarized in Table 1.

The genomic DNA of samples were genotyped with the Ovine Infinium HD SNP BeadChip (Illumina Inc., San Diego, CA, USA), which contains 606,006 SNPs spanning the whole ovine genome. The detailed criteria to control the quality of the whole genotype data are also followed as according to our previous study [14]. After filtering, 500,593 SNPs were available with a mean distance of 4.89 kb between adjacent SNPs, and 115 animals including 40 Altay sheep, 35 Large-Tailed Han sheep and 40 Tibetan sheep were retained for further analysis. The sporadic missing genotypes were imputed, and genotypes were phased using BEAGLE software [16].

### 2.3. Population Structure Analysis

To investigate genetic relationships between individuals and population, principal component analysis (PCA) was performed using the commands of make-grm and pca in GCTA software [17].

### 2.4. Selection Signature Detection Using hapFLK

In this study, hapFLK was performed to detect the selection signatures in three typical sheep population with obviously different tail types. The hapFLK proposed by Fariello et al. [18] is a haplotype-based approach applied to unphased genotypic data. This method was based on differences haplotype frequencies between populations using fastPHASE 1.4 to estimate the haplotype information. In this study, no outgroups were defined, 10 clusters (−K 10) were used for the fastPHASE model and the hapFLK statistic was computed for 20 EM runs to fit the LD model (–nfit = 20). The hapFLK 1.4.0 program version can be available at forgedga.jouy.inra.fr/projects/hapflk/files.

Since hapFLK values for each SNP on the entire genome approximately follow the normal distribution, hapFLK values were further standardized to calculate the *p*-values. The formula is as follows:

(1) $$hapFLK_{adj}=\frac{hapFLK-Mean(hapFLK)}{SD(hapFLK)}$$

where *Mean*(hapFLK) and *SD*(hapFLK) are the mean and standard error of all hapFLK values in the whole genome, respectively. The adjusted hapFLK follows a standardized normal distribution. Moreover, 0.01 significance level and Bonferroni correction were taken into account to calculate the genome-wide significance level. After adjustment, the threshold value was equal to −log10 (*p*-value) = 7.77 (0.01/500,593 independent tests).

### 2.5. Gene Annotation and Enrichment Analysis

Genes located on the selection regions were obtained from the Ensembl Genes 64 Database using BioMart software based on the Ovis aries (Oar_v3.1) gene sequence assembly. To learn the biological functions of annotated genes, we carried out a comprehensive literature search including information from other species.

## 3. Results

### 3.1. Population Genetic Structure

**Figure 2:**
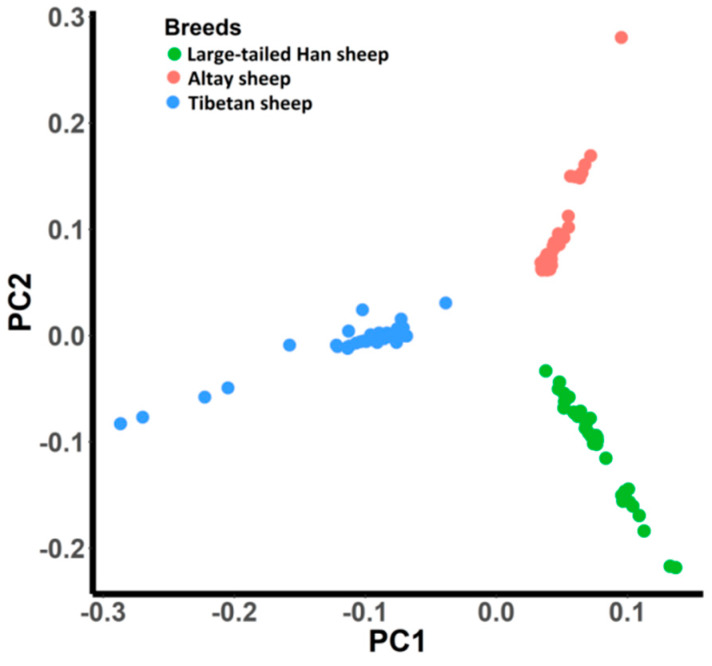
Principal component analysis (PCA) for population structure of three Chinese indigenous sheep breeds with extreme tail types. The first (PC1) and second (PC2) components were plotted.

PCA analysis uses all individuals (*n* = 115) and 500, 593 autosomal markers to assess whether all animals were divided into three groups adhere to different tail types. The first two principal components (PC1 and PC2) accounted for 3.3% and 2.7% of the variation, respectively, and divided all animals appropriate into three groups, as shown in Figure 2.

### 3.2. Identifying the Genomic Regions with Selection Signals Using hapFLK

**Figure 3:**
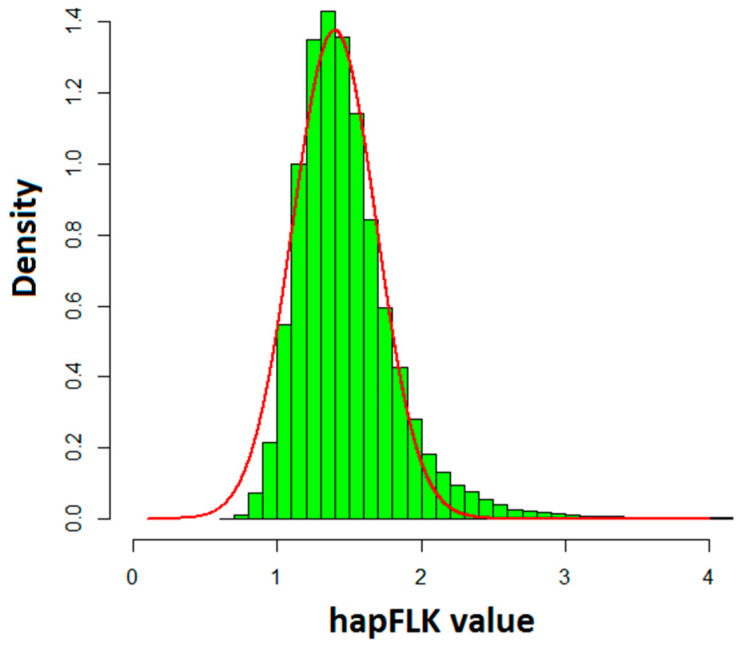
Distribution of hapFLK values over the sheep autosome. The histogram in green is the distribution of hapFLK values. The red line is the curve of normal distribution.

**Figure 4:**
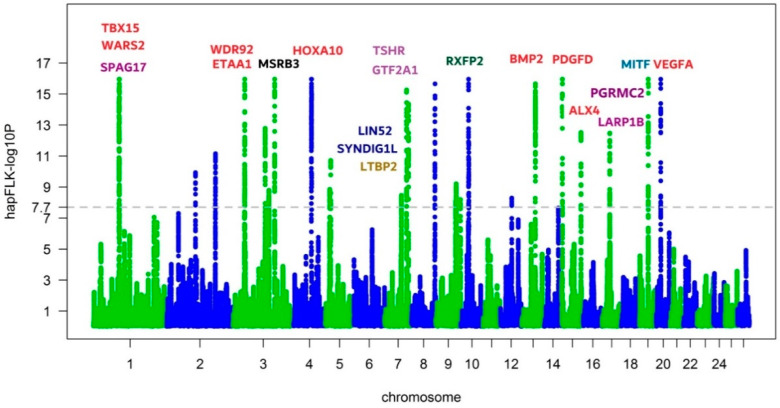
Manhattan plot of hapFLK values on the sheep autosome. The gray horizontal line denotes 0.01 genome-wide threshold (−log10(0.01/500,593) = 7.77).

The hapFLK statistic was implemented to find genomic regions under selection. As shown in Figure 3, the distribution of hapFLK values approximately follows the normal distribution. *p*-values for hapFLK calculated using an empirical method is appropriate because of ascertainment bias in the SNP panel [18]. Therefore, the *p*-value of each SNP was obtained from a normal distribution. Figure 4 shown the −log*p*-values in genome indicated that several regions suffered strong selection. To construct one genomic region with selection signals, the adjacent significant SNPs were grouped together.

### 3.3. Candidate Genes Annotated in Selection Regions

In total, we identified 25 genomic regions with selective signals, as shown in Table 2. The total length of genomic regions is 9.7 Mb. The largest length of genomic region is located on OAR10, while the genomic region on OAR1 contains the largest number of SNPs.

These genomic regions harbored 73 positional candidate genes. Out of these, six genes including *WDR92*, *BMP2*, *PDGFD*, *HOXA10*, *ALX4*, and *ETAA1* have been reported as implicated with tail formation in sheep [11,13,19,20]. Moreover, three genes (*VEGFA*, *TBX12*, and *WARS2*) have been associated with fat distribution in waist and hip in human [21], which indicate these genes may play important role in fat deposition in sheep tails.

Several genes were also identified to be associated with other economically important traits. *RXFP2* has been identified as being involved in horn morphology and was considered as the major effect gene in sheep [22,23]. *PGRMC2* and *SPAG17* have documented associated with fertility [24,25]. *TSHR* and *GTF2A1* genes are associated with photoperiod control of reproduction [26,27]. *LIN52* and *SYNDIG1L* were showed related to the number of teats in pig [28,29]. *LARP1B* is the primary candidate gene associated with milk fatty acids in dairy cattle [30]. *LTBP2* is a positional candidate gene for thoracic vertebrae in pigs [31]. *MSRB3* gene has shown to be the causal gene of ear size [32]. *JAZF1* can regulate lipid metabolism [33].

## 4. Discussion

Among multiple populations, common selection signatures can be detected using the fixation index (F_ST_). However, hidden population substructures exist in multiple breeds, which cannot be taken into account by F_ST_, due to its simplicity, and can lead to false positives. Moreover, these substructures are common in domestic animal populations. To address this limitation, a haplotype-based approach (hapFLK) was proposed that accommodates population substructures [18]. The hapFLK was implemented to detect selection signatures through haplotype differentiation among hierarchically structured populations. Hence, hapFLK can integrate the information from differences in haplotype allele frequencies among populations, the local haplotype data, and population substructures to identify genomic regions showing under selection. Furthermore, this approach can be applied unphased genotypic data and does not need information from the ancestral allele at each locus. Thus, hapFLK can be identified recent positive selection that results in a beneficial mutation residing on a haplotype with high frequency that is longer than average. In this study, we also employed the stringent significant threshold value (−log10(0.01/500935) = 7.7) to further control the positive signal. Therefore, the genomic regions undergoing selection would be more reliable.

In the present study, we selected Chinese indigenous sheep breeds with obviously different tail types. Large-Tailed Han sheep have the largest and fattiest tails in Chinese indigenous sheep breeds. Their tails sag below the hock and drag on the ground with circular shapes at the end of tails (Figure 1a). Altay sheep can deposit fat on the rump, which forms substantial buttocks (Figure 1b). This is another type of fat tail (fat-rumped). Meanwhile, Tibetan sheep have thin tails with a shape similar to that of a stick (Figure 1c). In addition, these three sheep breeds also live in a diverse range of ecological environments. Large-Tailed Han sheep are primarily distributed in the north Chinese plain, which has four distinctive seasons. Altay sheep live in the Gobi Desert, where the temperature ranges from −42.7 °C to +40.0 °C, and the ground is snow-covered for 200–250 days per year. Altay sheep can use the fat on their rumps to adapt to the harsh environment. Thin-tailed Tibetan sheep live in the Tibetan plateau at an altitude of 3000–5000 m. Therefore, in present study, we identified the candidate genes related not only to tail type formation but also to other economically important traits.

In the present study, we identified nine important candidate genes of tail formation in sheep (*WDR92*, *TBX12*, *WARS2*, *BMP2*, *VEGFA*, *PDGFD*, *HOXA*10, *ALX4*, and *ETAA1*). *WDR92* is a WD40 repeat protein that has several biological functions. Remarkably, as a WD40 repeat protein, WIPI1 has a seven-bladed propeller structure and encompasses a conserved motif to interact with phospholipids [34]. WIPI1 is also differentially expressed between adipocyte tissue from Kazak sheep’s and Tibetan sheep’s fat tails [35]. *ETAA1* plays an important role in fat distribution in fat-tailed sheep [11] and humans [36]. *WDR92* and *ETAA1* are also shown to be differentially expressed in the adipocyte tissue of sheep populations according to their different tail types [19]. The *BMP2* gene was shown to be differentially expressed in tail adipose tissue between fat-tailed and thin-tailed sheep using RNA-seq [20]. Members of the PDGF family promote proliferation and inhibit the differentiation of preadipocytes [19,20]. Thus, *PDGFD* played a pivotal role in sheep adipose tissue and is hypothesized to be one of candidate genes leading to fat tail formation. In the current study, *ALX4* was identified in the candidate region at chr15:72.5–72.6 Mb. Mutations are involved in the development of limbs and skeleton [6], and its protein has been shown to bind proteins from the *HOXA* clusters [37]. As one of *HOXA* cluster genes, *HOXA10* has showed to be differentially expressed between subcutaneous-, visceral- and tail-fat tissues by RNA-seq [38]. Moreover, other three genes (*VEGFA*, *TBX12*, and *WARS2*) have been associated with fat distribution in waist and hip in human [21], which indicate these three genes play important role in fat deposition in sheep tails. These findings indicate that these nine genes may be important candidate genes related to tail-type formation. Their functional verification needs further investigation and validation using full genome sequences and expression studies in the future.

To dissect the genetic basis of fat deposition in sheep, genome-wide association studies have also been performed directly using the fat-tail phenotypes and genotype data [39,40]. However, no common genes were identified by two strategies. This may be due to two main factors. One is the different principles of identifying candidate genes. Another is the use of different populations. However, the results by these two strategies suggest that the tail phenotype in sheep is most probably a complex trait.

In this study, the regions under selection contain other genes shown to be involved in agriculturally important traits. One of the most striking selective sweeps occurred at the locus for *RXFP2*, which has been shown to be associated with horn morphology and is considered as a major effect gene in sheep [22,23,41]. In the Large-Tailed Han sheep population, most males have spiral horns and most females have horns that are ginger-shaped or polled [2]. This gene was also identified by other studies on selection signature detection [6,10,42,43,44]. Furthermore, we found an interesting peak on OAR3:154.1~154.3 Mb harboring the *MSRB3* gene which is linked to ear size in sheep [10]. In the Altay population, few individuals have the small ear, and ear size morphologies exist in these three sheep breeds [2]. *MSRB3* was also identified as a candidate gene for ear morphology in dogs [45] and pigs [32]. Therefore, the *MSRB3* gene was hypothesized to be candidate gene for ear size in sheep. In line with a remarkable difference in coat color among these three sheep populations, we detected one potential candidate gene, *MITF*, known to be associated with pigmentation in sheep [5,46]. *LTBP2* was considered as a positional candidate gene for thoracic vertebrae in pigs [31]. *JAZF1* can regulate lipid metabolism [33] and is shown to be associated with height in human [47]. Moreover, this gene was also identified by selection signature in cattle [48]. The region on OAR7: 89.2~89.5 Mb harbored two important genes (*TSHR* and *GTF2A1*). The *TSHR* gene has a pivotal role in metabolic regulation and photoperiod control of reproduction in chicken [49] and also affects the seasonal reproduction of sheep and goats [50]. The *GTF2A1* gene is supposedly associated with the function of ovary and uterus and, hence, influenced egg production [27]. It is possible that selection for these variants could be associated with photoperiod control of reproduction in Chinese short fat-tailed sheep. *PGRMC2* and *SPAG17* have been documented as being associated with fertility [24,25]. *LIN52* and *SYNDIG1L* were shown to be associated with the number of teats in pig [28,29]. *LARP1B* is the primary candidate gene associated with milk fatty acids in dairy cattle [30]. These genes could be relevant for reproduction in these sheep.

## 5. Conclusion

We used Ovine Infinium HD SNP BeadChip genotype data to carry out a genome scan for selective sweeps in Chinese indigenous sheep breeds with extreme tail types. In total, 25 genomic regions were identified under selection which harbored 73 candidate genes. These genes are not only associated with tail fat formation but also with reproduction, body conformation, and appearance. Our findings contribute to the identification of candidate genes underlying important traits in different types of sheep breeds and provide a reference for developing new sheep breeds with an ideal tail type.

## Figures and Tables

**Table 1:** Description of phenotypic characteristics and environmental variables of three sheep breeds sampled in this study.

	Large-Tailed Han Sheep	Altay Sheep	Tibetan Sheep
Tail type	Long fat-tail	Fat-rumped	Long short-tail
No. individual	40	40	40
Body weight (average kg)	70.4 kg in male and 60.2 kg in female	98.3 kg in male and 77.1 kg in female	51 kg in male and 43.6 kg in female
Coat color	White	Brown	White body, heads and feet in miscellaneous colors
Horn	Most males have spiral horns and most females have ginger-shaped horns	Males have large horns, most females have horns	Males have large horns, females have horns
Region	Liaocheng county, Shan Dong Province	Fuhai county, Xinjiang Uighur autonomous region	Tianzhu county, Gansu Province
Geographical coordinates	N35°47′–37°02’ and E115°16´–116°32´	N40°00´–48°10´ and E87 °00´–89°04´	N36°31´–37°55´and E102 °07´–103°46´
Climate	Warm temperate continental monsoon climate	Continental arid climate	Continental plateau monsoon climate
Altitude (m)	22–49	386–3332	2040–4874
Temperature(°C)	−22.3–41.8	−42.7–40	−8–4
Use	Meat-fat dual type	Meat-fat dual and coarse wool type	Coarse wool type

Note: The data were derived from animal genetic resources in China for sheep and goats [2].

**Table 2:** Genes located in genomic regions showing evidence of selection.

Chr	Start	End	No. SNP	Size (kb)	Candidate Genes
1	94756172	96112605	224	1356.433	*SPAG17*, *RF00026*, *TBX15*, *WARS2, RF00100, HAO2, HSD3B1*
2	105901381	105961277	11	59.896	*-*
2	183012215	183196527	34	184.312	*-*
3	39961457	40797601	172	836.144	*PNO1, WDR92, C1D, ETAA1*
3	118322493	118505234	38	182.741	*METTL25*, *RF00001*
3	132458013	132563310	26	105.297	*-*
3	154055122	154324519	58	269.397	*MSRB3*
4	68223979	69165823	183	941.844	*JAZF1, TAX1BP1, EVX1, RF00275, RF01975, RF01976, RF01978, RF01979, RF02040, RF02041, RF02042, RF02043, RF02137, RF02138, RF02139, RF02140, RF02141, RF02142, RF02143, HOXA1, HOXA2, HOXA3, HOXA6, HOXA10, SKAP2*
5	16293532	16380709	19	87.177	*ASFB2*
5	20143416	20250955	12	107.539	*-*
7	63493868	63648665	16	154.797	*-*
7	82373886	82795821	89	421.935	*LIN52, VSX2, ABCD4, SYNDIG1L, NPC2, RF00004, LTBP2*
7	89260877	89527917	51	267.04	*TSHR, GTF2A1, RF00600*
8	87547094	88056486	108	509.392	*TBXT, MPC1, RPS6KA2*
9	75623521	75637947	2	14.426	*-*
9	77403165	77840673	47	437.508	*VPS13B*
9	93544798	93558759	3	13.961	*SLC10A5*, *ENPP2*
10	29320784	30719200	88	1398.416	*RXFP2*, *B3GLCT*, *HSPH1*, *TEX26*, *MEDAG*, *ALOX5AP*, *USPL1*
12	42361453	42380380	8	18.927	*H6PD*
13	48400700	49177821	81	777.121	*BMP2*
15	3434222	3945775	68	511.553	*PDGFD*
15	72489270	72637084	28	147.814	*EXT2, RF00026, ALX4*
17	29091651	29420825	44	329.174	*PGRMC2, LARP1B*
19	31539577	31850022	64	310.445	*MITF*
20	17281001	17555177	44	274.176	*VEGFA*
